# Supplementary material for: Effects of Adenotonsillectomy on Vocal Function
Source: J Pers Med. 2023 Jun 15;13(6):1002. doi: 10.3390/jpm13061002 (PMC10301818; doi:10.3390/jpm13061002)
Supplement: Supplementary file 1 [file jpm-13-01002-s001.zip › jpm-2390375-supplementary.pdf]

Investigation protocol

Name and surname:____________________________ Phone number:_______________

Date of birth: ___/___/_______ Age:______ Gender: ☐ M ☐ F

Speech evaluation:

Reported respiratory mode: ☐ Oral ☐ Nasal ☐ Mix

Reported respiratory difficulties:

- Allergies: ☐ Yes ☐ No
- Nocturnal snoring: ☐ Present ☐ Absent
- Others: ____________________________________

Oral posture: ☐ Closed ☐ Semi-closed ☐ Open

Subjective symptoms: ____________________________________

Objective symptoms

Phonics chart

Pitch level:

Elevated

Regular

Reduced

Inconsistent

Vocal Intensity:

Elevated

Regular

Reduced

Inconsistent

Vocal timbre:

Hoarse

Personal (Specific to the subject)

Veiled

Inconsistent

Phonetic attack:

Hard

Correct

Delayed

Inconsistent

Vocal hold:

Regular

Reduced

Phonation duration:

Regular

Reduced

<u>Respiratory framework:</u>

Thoracic-apical

Costo-diaphragmatic

Mix

Closed nasality: □ Present □ Absent

- Phonetic balance:

i	e<br>ɛ	a	o	u

p	b
t	d
k	g

f	v
s	z
ʃ	

ts	dz
tʃ	dʒ

m
ɱ
n
ŋ
ɲ

l	r
ʎ	

On repetition

Stable phonemes:

Occasional phonemes:

Absent phonemes:

System simplifications:

Structure simplifications:
